# Oxytocin and secretin receptors – implications for dry eye syndrome and ocular pain

**DOI:** 10.3389/fopht.2022.948481

**Published:** 2022-08-01

**Authors:** Jacqueline B. Lopez, Chih-Chiun Chang, Yien-Ming Kuo, Matilda F. Chan, Bryan J. Winn

**Affiliations:** ^1^ Department of Ophthalmology, University of California, San Francisco, San Francisco, CA, United States; ^2^ Department of Ophthalmology, Zuckerberg San Francisco General Hospital and Trauma Center, San Francisco, CA, United States; ^3^ Francis I. Proctor Foundation, University of California, San Francisco, San Francisco, CA, United States; ^4^ Surgical Service, San Francisco Veterans Affairs Medical Center, San Francisco, CA, United States

**Keywords:** oxytocin, secretin, ocular pain, anti-inflammatory, dry eye syndrome

## Abstract

Dry eye syndrome, a form of ocular surface inflammation, and chronic ocular pain are common conditions impacting activities of daily living and quality of life. Oxytocin and secretin are peptide hormones that have been shown to synergistically reduce inflammation in various tissues and attenuate the pain response at both the neuron and brain level. The oxytocin receptor (OXTR) and secretin receptor (SCTR) have been found in a wide variety of tissues and organs, including the eye. We reviewed the current literature of *in vitro* experiments, animal models, and human studies that examine the anti-inflammatory and anti-nociceptive roles of oxytocin and secretin. This review provides an overview of the evidence supporting oxytocin and secretin as the basis for novel treatments of dry eye and ocular pain syndromes.

## Introduction

Dry eye syndrome (DES) affects up to 15% of the adult population, and up to 30% of patients over age 50, causing significant impairment in visual acuity, workplace functioning, and activities of daily living (1, 2). Originally thought to be a condition of reduced tear volume, subsequent research shows that the pathogenesis involves a complex interplay of multiple autoimmune, inflammatory, hormonal, and pain pathway influences (3, 4). Despite a better understanding into the mechanism of dry eye pathogenesis, currently available treatments are limited to symptomatic relief with artificial tears and non-specific immunosuppressive medications (5).

Oxytocin is a nine amino-acid neuropeptide hormone synthesized in the paraventricular and supraoptic nuclei of the hypothalamus and has well-studied roles in parturition, lactation, social bonding, and neuropsychological disorders (6). It binds to the oxytocin receptor (OXTR), which is a member of the rhodopsin-type (class I) G-protein coupled receptor family that contains seven transmembrane α-helices and is coupled to Gα_q/11_, Gα_i/o_, or Gα_s_ heterotrimeric complexes (7–10). When coupled to Gα_q/11_ class GTP binding proteins, stimulation of this receptor activates phospholipase Cβ (PLCβ) catalyzing the hydrolysis of phosphatidylinositol 4,5-bisphosphate (PIP_2_) to generate inositol triphosphate (IP3) and 1,2-diacylglycerol (DAG), which in turn leads to intracellular calcium release and protein kinase C stimulation, respectively (11). In conjunction with calmodulin, intracellular calcium then activates protein kinases involved in myoepithelial cell contraction. While the oxytocin PLC/Ca2+ pathway is well known for its role in mammary gland and smooth muscle function, it has recently been shown to induce lacrimal gland myoepithelial cell contraction (7). OXTR can also be coupled to Gα_i/o_, resulting in inhibition of adenylate cyclase and a decrease of intracellular cyclic adenosine monophosphate (cAMP) (10). OXTR coupled with Gα_s_ potentially increases cAMP; however, its physiologic existence is questionable with only low or insignificant coupling ever demonstrated (8–10). Depending on the tissue type, stimulation of OXTRs coupled with Gα_q/11_ or Gα_i/o_ complexes can act synergistically, as in the case of myometrial cell contraction, or antagonistically as occurs with inward rectifier K ^+^ channel conductance in olfactory neurons (12) and cell growth in human embryonic kidney cells (13–15).

The OXTR can also signal *via* G protein-independent pathways. Stimulation of the OXTR and subsequent phosphorylation by G protein coupled receptor kinases induce the recruitment of β-arrestins (9, 16). While β-arrestins lead to desensitization of the OXTR, they also have been shown to simultaneously activate downstream signaling, including the mitogen-activated protein kinase (MAPK) pathway (16).

OXTR has been shown to be expressed in the tongue, nose, retina, gut, and brain (11, 17–20). Studies suggest anti-inflammatory roles for oxytocin in the heart (21), skin (22), and gastrointestinal tract (23), as well as pain mitigation in the setting of chronic low back pain (24), facial pain syndromes (25), and migraine headaches (26, 27).

The secretion of oxytocin and expression of OXTR have been shown to be influenced by expression of secretin, a peptide hormone that regulates water homeostasis and pancreatic secretions, and the secretin receptor (SCTR) in the hypothalamus (28, 29). Rodent model studies of inflammatory bowel disease demonstrate that oxytocin works synergistically with secretin to reduce inflammation in the gut (30). These results suggest that dual peptide treatment may modulate inflammation in tissues and organs that express both OXTR and SCTR and that secretin may potentiate the action of oxytocin. The presence of OXTR has been previously described in the retina, where it was proposed to have a role in paracrine signaling with the retinal pigment epithelium (19). OXTR had also been identified in human and mouse lacrimal glands with its expression decreased in the setting of dry eye mouse models (31). Additionally, OXTR and SCTR have been shown to be expressed on the corneal surface of humans and rats, although their function is unknown (32). Given their anti-inflammatory roles and presence on relevant ocular structures, these neuropeptide pathways potentially may be harnessed for novel treatments of dry eye and ocular pain syndromes.

PubMed was used to search for articles containing combinations of keywords: (oxytocin OR secretin) AND (inflammation OR anti-inflammatory OR pain OR nociception OR trigeminal neuralgia OR eye OR dry eye syndrome OR Sjögren’s syndrome). Available articles in English were reviewed and included in this review if found to be relevant to the anti-inflammatory or anti-nociceptive effects of OXTR and/or SCTR on the eye.

## Dry eye syndrome and ocular pain

Chronic ocular pain is a significant component of DES that may be less responsive to conventional treatment including lubrication, topical corticosteroids, and immunomodulators (33). Multiple studies suggest that DES patients can experience symptoms consistent with neuropathic pain, including hypersensitivity to environmental triggers (wind, light, heat), spontaneous burning unresponsive to therapies, severe symptoms with minimal exam findings, co-existing psychologic distress, and increased pain sensitivity in regions other than the eye (34, 35). Patients with increased chronic pain-related syndromes exhibit more severe neuropathic-type dry eye symptoms, suggesting that a subset of dry eye patients resistant to conventional treatment may manifest an underlying central pain processing disorder (36).

There are several proposed pathways that modulate neuropathic pain. Nerve growth factor (NGF), released in chronic inflammation, increases sensitivity to painful stimuli by activating peripheral nociceptors *via* tyrosine kinase (trkA) receptors (37). A second proposed pathway is mediated by corneal nociceptors that transmit pain signals along the trigeminal nerve (V1) followed by the release of glutamate binding to AMPA (α-Amino-3-hydroxy-5-methyl-4-isoxazolepropionic acid) receptors for the sensation of acute pain and NMDA (N-methyl-D-aspartate) receptors for chronic pain, resulting in a continued transmission of pain signals to higher-order neurons (37). Additionally, in one genome-wide analysis of differentially expressed genes following dorsal root ganglion compression, several key genes that may play a role in neuropathic pain development were found, including syndecan 1 (Sdc1); phosphatidyloinositol-4,5-bisphosphate 3-kinase, catalytic subunit gamma (Pi3k); Janus kinase 2 (Jak2); Jun proto-oncogene, AP-1 transcription factor subunit (Jun); and interleukin 6 (IL-6) (38).

In addition to neuropathic pain, there is also a component of nociceptive pain in DES. Heightened sensitivity to pain and diminished pain tolerance are significantly associated with symptoms of dry eye disease (39). This type of pain involves several nociceptor pain fibers, such as transient receptor protein (TRP) channels, that are responsible for sensing tissue inflammation and damage (40). Environmental triggers can lead to damaged epithelial cells and corneal nerves, causing an upregulation of pro-inflammatory mediators within the cornea of dry eye patients. These inflammatory stimuli include prostaglandin 2 (PGE2), cyclooxygenase 2 (COX2), various interleukins (IL-2, IL-4, IL-5, IL-6, IL-8, IL-10, IL-17), tumor necrosis factors alpha (TNF-α), reactive oxygen species, nerve growth factor (NGF), and metalloproteinase-9 (MMP-9) (40). Pain hypersensitivity and central sensitization involving the trigeminal brainstem may result from persistent ocular surface damage, inflammation, and pain nerve stimulation, leading to chronic dry eye symptoms that are disconnected from the pathology seen in the eye (40, 41). Patients with greater dry eye and reported ocular pain symptoms also display hypersensitivity within corneal somatosensory pathways that suggest modulation of specific nerve pathways (42). Furthermore, dry eye symptoms have been correlated with measures of anxiety and pain sensation at distant locations in the forearm, suggesting diffuse somatosensory dysfunction beyond the trigeminal system (43).

## Oxytocin’s role in pain reduction

It is well-documented that neuropathic pain similar to symptoms experienced by patients with DES is mediated by chronic inflammation in neurons and increased excitability in brain regions associated with somatosensory processing and stress (44). **Table 1** outlines the anti-nociceptive roles of oxytocin and secretin. There is evidence from rat models that oxytocin modulates pain processing at the rostral angular insular cortex, a brain structure important in nociception (45). At the level of the spinal cord, animal models of neuropathic pain have shown that oxytocin inhibits nociceptive neurotransmission at the medullary dorsal horn by suppressing transmission from sensory Aδ and C-fibers (54) and increasing GABAergic inhibitory transmission to TRPV1 (transient receptor potential channel vanilloid 1), a non-selective cation channel that transmits painful stimuli in the nervous system (46). In addition, oxytocin suppresses the expression of inflammatory cytokines TLR-4, TNF-α, and IL-1β while down-regulating mechanical and thermal pain sensation in rat models (47). At the neuronal level, oxytocin inhibited activation of the trigeminocervical complex in animal models (48) by blocking the release of calcitonin gene-related peptide, a neuropeptide that regulates nociceptive signaling (27). The mechanism of oxytocin’s effects may be mediated by increasing intracellular chloride concentrations with the KNCC1 chloride co-transporter and depolarizing GABA on nociceptive trigeminal neurons (49) or increasing voltage-gated K+ channel currents in the trigeminal ganglia, decreasing the resting potential and activity of the neurons (25). One study showed that oxytocin was effective in alleviating vincristine-induced neurite damage in cultured primary dorsal root ganglion neurons *in vitro* and vincristine-induced hyperalgesia with an *in vivo* mouse model (50).

**Table 1:** Anti-nociceptive roles of oxytocin and secretin.

Paper Cited	Hormone(s)	Model Organism	Anatomical Site	Proposed Mechanism	Physical Findings
Kubo et al. (25)	Oxytocin	Rat – partial ligation of infraorbital nerve	Oxytocin applied to trigeminal ganglion (TG)	Increased voltage-gated K channel currents ➔ suppressed TG neuronal hyperexcitability after nerve injury	Reduced mechanical hypersensitivity in whisker pad skin
Tzabazis et al. (27)	Oxytocin	Rat	Trigeminal ganglion (TG)	Decreased capsaicin-induced calcitonin gene-related peptide release from dural nociceptors	Electrocutaneous stimulation and adjuvant-induced inflammation ➔ upregulation of oxytocin e-receptor protein expression in TG neurons
Gamal-Eltrabily et al. (45)	Oxytocin	Rat	Rostral agranular insular cortex (RAIC)	Modulates pain processing at cortical insular level by increasing cortical GABAergic transmission and activating descending spinal noradrenergic mechanisms	Oxytocin microinjection into RAIC during inflammatory nociceptive input (formalin injection) reduced flinches and spontaneous firing of spinal wide dynamic range cells Effect abolished by OXTR antagonist and GABAA receptor blocker; partially reversed by α2A-adrenoreceptor antagonist
García-Boll et al. (2017)	Oxytocin	Rat	Medullary dorsal horn (MDH)	Dose-dependently inhibits peripheral-evoked activity in nociceptive MDH neurotransmission – associated with blockade of Aδ- and C-fibers Effect abolished by OXTR antagonist, not affected by vasopressin V1A receptor antagonist	
Sun et al. (46)	Oxytocin	Rat – partial ligation of sciatic nerve	Sciatic nerve/spinal cord	Relieves neuropathic pain through GABA release and presynaptic TRPV1 inhibition in spinal cord	Alleviated mechanical allodynia and thermal hyperalgesia in partial sciatic nerve ligation Inhibits capsaicin-induced ongoing pain in rats
Mou et al. (47)	Oxytocin	Rat – bone cancer pain model	Spinal cord (intrathecal)	Suppresses up-regulation of TLR4, IL-1β, TNF-α in spinal cord	Ameliorates mechanical allodynia and thermal hyperalgesia
García-Boll et al. (48)	Oxytocin	Rat	Trigeminocervical complex (TCC)	Reduced TCC neuronal firing evoked by meningeal electrical stimulation OXTR antagonist abolished this effect	
Mazzuca et al. (49)	Oxytocin	Rat – newborn pups		Reduces depolarizing action of GABA on nociceptive neurons	Reduced pain in newborn and 2-day pups (measured by thermal tail-flick assay, electrical whisker pad stimulation) OXTR antagonist enhanced pain sensitivity in newborn pups
Zhu et al. (50)	Oxytocin	Mice – vincristine (VCR)-induced neurotoxicity; OXTR-knock out (KO) Cultured primary dorsal root ganglion (DRG) neurons	Dorsal root ganglion, sciatic nerve		Alleviated VCR-induced hyperalgesia Attenuated VCR-induced damages of nerve endings, myelin sheaths, Schwann cells in sciatic nerve and DRG Effects diminished with OXTR antagonist OXTR-KO mice showed more severe hyperalgesia than wild-type
Meidahl et al. (51)	Oxytocin	Rat – mild traumatic brain injury (TBI) model	Brain, trigeminal nerve	Reduced mild TBI pain by binding to OXT or VA1-receptors, most likely by peri-trigeminal nerve mediated uptake	Attenuated reactive and spontaneous, ongoing non-reactive pain following mild TBI for 3-4 hours after intranasal administration
Hu et al. (52)	Oxytocin Receptor (OXTR)	Rat	Lateral (CeL) nucleus of central amygdala	Activation of OXTR increased action potential firing frequency in CeL neurons *via* inhibition of inwardly rectifying K+ channels Required phospholipase Cβ and protein kinase C to increase neuronal excitability	
Ando et al. (53)	Oxytocin	Rat – infraorbital nerve injury (IONI)	OXT application to infraorbital nerve injury site	Inhibits increase in transient receptor potential vanilloid 1(TRPV1)-IR and TRPV4-IR TG neurons ➔ attenuates post-IONI orofacial mechanical allodynia	

In several animal models, oxytocin attenuates trigeminal (26, 51) as well as visceral pain (51). At the neurophysiologic level, oxytocin receptors have been detected in the lateral capsular division of the central nucleus of the amygdala, with anxiolytic effects mediated by the depression of inwardly rectifying K+ channels (52, 55). Activation of parvocellular oxytocin neurons in the paraventricular nuclei can also inhibit spinal pain processing by repressing nociceptive transmission from Aδ and C-type afferent signaling pathways (56). In a rat model of neuropathic pain, oxytocin application to nerve-injured sites decreased the amount of TRPV1-immune reactive trigeminal neurons, key players in nociception, that innervated the whisker pad skin. This led to an attenuation of orofacial mechanical allodynia following infraorbital nerve injury (53). In another rat study of neuropathic pain involving infraorbital nerve injury, the combined effects of oxytocin application to the locally injured nerve and low-level laser therapy showed a decrease in cortical excitation in response to electrical stimulation of dental pulp, reflecting a reduction of neuropathic pain processed by the animals’ brains (57). In animal models of intestinal inflammation, the combination of secretin and oxytocin decreases the excitability of central neurons located in the hypothalamus, amygdala, and piriform cortex, areas of the brain critical for the emotional processing of pain (30), with similar findings in the frontal cortex, hippocampus, thalamus, and midbrain observed in separate models (26). In a double-blinded, placebo-controlled, cross-over design trial involving 30 healthy human subjects, a single dose of 40 IU/ml intranasal oxytocin was shown to enhance endogenous pain inhibition in the trigeminal distribution *via* conditioned pain modulation as well as improved mood (58). Another double-blinded, placebo-controlled trial of ten individuals with migraine showed a dose-dependent response of oxytocin in reducing headache pain with oxytocin levels positively correlated with measures of symptom severity (59).

## Anti-inflammatory effects of oxytocin and secretin

Ocular surface inflammation is an important risk factor for and subsequent consequence of DES (3). Both the innate and adaptive immune system play key roles in the pathogenesis of DES. The innate immune response in DES has been shown to involve activation of interleukin and tumor necrosis factors through the MAPK pathway and upregulation of immune cells through Toll-like receptor signal transduction (60). The adaptive immune response in DES has been shown to involve CD4+ T cells and antigen presenting cells (APCs), which increase production of a multitude of cytokines, including IFN-gamma, IL-6, IL-17, IL-4, and IL-1, that contribute to further pro-inflammatory pathways, such as NF-κB and corneal epithelial caspase-1 pathways (60).

There is evidence supporting the anti-inflammatory roles of oxytocin and secretin in multiple organ systems, outlined in **Table 2**. Oxytocin has been shown in rodent models to decrease plasma concentrations of cytokines including TNF-α, IL-1, IL-4, and IL-6 in the setting of bacterial infection (61), wound healing (62), and intestinal inflammation (30, 63), where there was synergy with secretin. Rodent model studies suggest that oxytocin has widespread effects on leukocyte activation, expression, and migration, while decreasing stress-induced release of reactive oxygen species and corticosterone (64, 74–76). In an obesity mouse model, the administration of oxytocin decreased TNF-α secretion and the macrophage M1/M2 ratio, inducing an anti-inflammatory phenotype with improvements in glucose tolerance (65). In another obesity mouse model study, expression of adipose tissue OXTR was increased in obese mice compared to lean controls. Exposure to long-term oxytocin treatment led to reductions in visceral adipose tissue inflammation, with decreased adipocyte size, macrophage infiltration, IL-6 and TNF-α expression. Adiponectin, a key anti-inflammatory adipokine, was increased in both plasma and adipose tissue, while plasma levels of serum amyloid A, a marker of systemic inflammation, were decreased (66). *In vivo* and *in vitro* models of food allergy showed that oxytocin inhibits the production of multiple cytokines, including thymic stromal lymphopoietin (TSLP), IL-33, and IL-25, through suppression of NF-κB signaling and upregulation of B-arrestin2 expression, which decreased systemic anaphylactic responses and intestinal inflammation in mice (67). In a separate inflammatory bowel disease (IBD) murine model, OXTR knock-out mice displayed an increased number of pro-inflammatory cytokine transcripts encoding for TNF-α, IL-1β, and IL-6 and exhibited shorter villi and crypts in the intestinal mucosa, a sign of chronic inflammation. In addition, colonic tissue in OXTR knock-out mice was more susceptible to cholera toxin-induced inflammation compared to a blunted immune response observed in wild-type mice treated with oxytocin (68). Oxytocin-treated mice also had a reduction in neutrophils and macrophages observed in the mucosal and submucosal layers of the colon and decreased expression of TNF-α and IFN-γ, leading to clinical improvement in ulcerative disease (30). At the cellular level, *in vitro* experiments using gut epithelial cells have demonstrated that oxytocin modulates important molecules in the stress signaling pathway (69, 77) and plays a regulatory role in translation of proteins (69). In addition, oxytocin may protect epithelial cells from inflammation (70). Mouse models of blepharitis, Sjögren’s keratoconjunctivitis sicca, and DES have demonstrated elevated levels of inflammatory cytokines, including IL-1, IL-6, and TNF-α (78–80) although the role of oxytocin in modulating these inflammatory cytokines on the ocular surface is currently unknown.

**Table 2:** Anti-inflammatory roles of oxytocin and secretin.

Paper Cited	Hormone(s)	Model Organism	Anatomical Site	Molecular Effects	Physical/Pathology Findings
Nation et al. (21)	Oxytocin	ApoE-/- Mice	Heart	Reduced IL-6	Decreased atherosclerosis
Petersson et al. (22)	Oxytocin	Rat	Skin	Reduced myeloperoxidase (marker of neutrophil recruitment)	Reduced edema
Iseri et al. (23)	Oxytocin	Acetic acid-induced colitis in rat	GI tract	Reduced malondialdehyde (end-product of lipid peroxidation), myeloperoxidase (MPO, index of neutrophil infiltration) in colon tissue Reduced LDH and TNF-α	Reduced acetic acid-induced colonic fibrosis
Welch et al. (30)	Secretin and Oxytocin	Colitis in rat	Colon	Reduced TNF-α and IFN-γ in colon tissue	Decreased colonic inflammatory infiltrates
Hawley et al. (2021)	Oxytocin	Aqueous deficient dry eye in NOD and MRL/lpr mice; humans	Lacrimal glands, myoepithelial cells (MECs)		OXTR is highly expressed in MECs of mouse and human lacrimal glands – reduced expression in diseased glands Oxytocin mediates contractions of lacrimal gland acini – impaired in diseased glands
Clodi et al. (61)	Oxytocin	Bacterial endotoxinemia in humans	Plasma	Reduced endotoxin-induced plasma ACTH, cortisol, procalcitonin, TNF-α, IL-1 receptor antagonist, IL-4, IL-6, macrophage inflammatory protein-1α, macrophage inflammatory protein-1β, monocyte chemoattractant protein-1, interferon-inducible protein 10, VEGF	
Poutahidis et al. (62)	Oxytocin	Oxytocin-KO mice	Skin and mucosal tissues		Worsened skin wound repair in OXT-KO mice (delayed re-epithelialization, delayed collagen and fibrinogenesis, increased accumulation of neutrophils)
Cetinel et al. (63)	Oxytocin	Colitis and stress in rat	Colon	Reduced MPO, MDA	Decreased colitis-induced anxiety Reduced inflammatory cell infiltration and submucosal edema
Inoue et al. (2017)	Oxytocin	Mice	Brain – microglia	Inhibits eIF-2α-ATF4 pathway ➔ suppresses TNF-α, IL6, IL-1β	
Wang et al. (64)	Oxytocin	Autistic mice (valproate-induced)	Brain – hippocampus, amygdala, microglia	Reduced Il-1β, IL-6, TNF-α in hippocampus and amygdala Reduced microglia activation in hippocampus, amygdala	Improves anxiety, depression, repetitive behavior, and social interactions
Garrido-Urbani et al. (65)	Oxytocin	Diet-induced obese mice	Bone marrow cells Adipose tissue	Decreased TNF-α secretion in M1-derived macrophages Decreased TNF-α in adipose tissue	Induced anti-inflammatory phenotype with decreased M1/M2 macrophage ratio Decreased body weight, improved glucose tolerance
Szeto et al. (66)	Oxytocin	Obese mice	Adipose tissue Plasma	Reduced IL-6 and TNF-α, increased adiponectin (anti-inflammatory marker) in adipose tissue Increased adiponectin, decreased serum amyloid A (inflammatory marker) in plasma	Reduced decreased adipocyte size and macrophage infiltration
Yu et al. (67)	Oxytocin	Food allergy in mice	GI tract	Inhibits thymic stromal lymphopoietin, IL-25, IL-33	Decreased systemic anaphylactic response and intestinal inflammation
Welch et al. (68)	Oxytocin	IBD/OXTR-KO mice	GI tract	OXTR-KO ➔ increased TNF-α, IL-1β, IL-6	OXTR-KO ➔ shorter villi and crypts in intestinal mucosa
Klein et al. (69)	Oxytocin	Caco2BB human gut cell line	Gut cells	Biphasic response in PI3k/Akt pathway, activation peaks with OXTR internalization	
Klein et al. (70)	Oxytocin	Caco2BB human gut cell line	Gut cells	Suppresses NF-kB signaling and counteracts LPS-elicited silencing of the unfolded protein response Activates dsRNA-activated kinase, X-box binding protein 1, immunoglobulin binding protein, A20 (TNF-α-induced protein 3), inositol requiring enzyme 1a Inactivates eukaryotic translation initiation factor 2a	
Spangelo et al. (71)	Oxytocin	Neurointermediate Pituitary lobe (NIL) primary cell culture from rat tissue	NIL tissue from pituitary glands	Inhibited LPS and IL-1β stimulation of IL-6 release from NIL cells	
Yang et al. (72)	Oxytocin	Human – with dry eye disease, nebulized OXT treatment	Ocular surface		Improved Ocular Surface Disease Index scores, light sensitivity, dryness, tear meniscus height Increased basal epithelial cell density, decreased dendritic cell density, increased sub-basal nerve density and tortuosity
Gilbard et al. (73)	Secretin	Rabbit – keratoconjunctivitis sicca model	Eye		Decreased tear film osmolarity – effect was blocked by prior administration of proparacaine Increased tear secretion by irritative sensory stimulation (blocked when ocular surface is anesthetized)

## Synergism between secretin and oxytocin

There is evidence of synergistic activity between secretin and oxytocin, an interaction that may enhance their usefulness as anti-inflammatory agents. Indeed, the release of oxytocin is directly influenced by the levels of circulating secretin through stimulation of α1-adrenoreceptors and SCTR (81). Secretin has been shown to activate supraoptic oxytocin neurons, which express SCTR, and facilitate release of oxytocin from these nerve dendrites in mice (28). In rats, secretin can increase the firing rate of supraoptic oxytocin neurons through noradrenergic pathways, thereby increasing the plasma concentration of oxytocin (82). In a rat model of chronic colitis, combined intravenous (IV) administration of secretin and oxytocin led to decreased inflammatory infiltrates in the colon and reduced expression of TNF-α and IFN- γ in colonic tissue. Interestingly, IV administration of oxytocin or secretin alone did not produce a significant change in colonic inflammation. Combined IV administration of oxytocin and secretin also inhibited colitis-associated activation of forebrain neurons in the paraventricular nucleus of the hypothalamus, basolateral amygdala, central amygdala, and piriform cortex (30).

## Oxytocin and secretin’s roles in dry eye syndrome

Oxytocin and OXTR are expressed in the myoepithelial cells of the lacrimal gland, stimulating the contraction of acinar cells to secrete tears. The number of oxytocin receptors and myoepithelial cells are significantly reduced in mouse models of Sjögren’s syndrome, suggesting oxytocin’s role in maintaining tear production. Stimulation of oxytocin receptors in the lacrimal glands of healthy, control mice resulted in contraction of acini and the production of tears that was not present when dry eye disease mice were exposed to oxytocin (31). The pro-inflammatory state of DES may also be driven by corneal epithelial dysfunction and increased sensitivity to bacterial metabolites produced by commensal organisms on the ocular surface. For example, in dry eye mouse models, bacterial lipopolysaccharides (LPS) increases the expression of inflammatory mediators including IL-1β, IL-6, CXCL10, IL-12a, and IFN-γ in the conjunctiva and IL-1β and CXCL10 in the cornea (83). *In-vitro* studies of anterior pituitary cell culture provided the first evidence that oxytocin can inhibit LPS and IL-1β stimulation of macrophages, T-cells, and B-cells and decrease IL-6 cytokine production (71).

Animal studies have demonstrated that secretin increases tear production but not when topical anesthesia is applied, suggesting a sensory-dependent mechanism of effect (73). Vasoactive intestinal peptide (VIP) and pituitary adenylate cyclase-activating polypeptide (PACAP), which are part of the same superfamily of structurally related peptide hormones that includes secretin, glucagon, glucagon-like peptides, gastric inhibitory peptide (GIP) and growth hormone-releasing hormone (GHRH), have been shown to increase tear production by upregulating cyclic adenosine monophosphate (cAMP) and cyclic guanosine monophosphate (cGMP) production and protein kinase A phosphorylation, resulting in downstream aquaporin protein expression stimulating fluid and protein flow (73, 84, 85).

## A role for topical oxytocin and secretin in dry eye syndrome and ocular pain?

Animal and human studies suggest that oxytocin can be administered safely for local treatment with little to no systemic side effects. In mouse, rabbit, and primate models, there was minimal systemic absorption of oxytocin through the nasal mucosa (86, 87) and ocular surface (88). In a mouse model of mild traumatic brain injury (TBI), intranasal oxytocin application led to an attenuation of reactive and ongoing non-reactive pain following mild TBI for at least 3-4 hours. Interestingly, IV administration of oxytocin did not have the same pain attenuating effects, and there were higher concentrations of oxytocin found in the trigeminal ganglion following intranasal application compared to IV administration of oxytocin (51). A 20-year review of intranasal oxytocin use in human research studies found that dosages of 18-40 IU oxytocin had no reliable side effect profile compared to placebo and no reported adverse outcomes (89).

Several analogs of oxytocin, including lipo-oxytocins and TGOT, have been studied in the basic science and clinical trial contexts for social anxiety, autism spectrum, and other neuropsychiatric disorders, and shown to be powerful agonists on OXTR (90–92). Carbetocin is another oxytocin analog that has been extensively studied as treatment for postpartum hemorrhage when peripherally administered (92, 93). Unlike oxytocin itself, recent evidence points to oxytocin analogs having differential abilities to activate the OXTR, and subsequent downstream effects, depending on which G protein with the OXTR is coupled (15), raising the potential of selective OXTR-based therapies. Recently, a non-peptide agonist of oxytocin receptor (LIT-001) was able to induce a durable reduction in inflammatory pain-induced hyperalgesia in a rat model (94). Several small molecules have shown evidence as allosteric modulators of the SCTR, expanding potential drug options beyond secretin and its peptide analogs (95, 96).

There has been evidence for oxytocin and secretin’s role in reducing inflammation within human corneal cells. In a cell culture model of ocular surface inflammation created through the exposure of TNF-α to human corneal cells, levels of ICAM-1 expression were measured following administration of oxytocin and/or secretin. Three hours after addition of oxytocin or secretin, ICAM-1 levels were reduced about 40% compared to TNF-α treatment alone. A combination treatment of both oxytocin and secretin did not result in further reduction of ICAM-1 levels. The effect of the hormonal treatment was transient, with a maximal change at 3-4 hours (32).

A recent prospective cohort study investigated the use of nebulized oxytocin compared to vitamin B12 for the treatment of dry eye disease. Thirty-eight patients with DES were enrolled, with half receiving oxytocin (OXT) nebulization and the other half receiving vitamin B12 (VB12) nebulization treatment twice weekly for 3 months. Several clinical measurements were taken at baseline, 1 month, and 3 months after starting treatment, including Ocular Surface Disease Index (OSDI) questionnaire, self-reported light sensitivity and dryness, tear meniscus height (TMH), tear break-up time (BUT), and corneal staining. *In vivo* confocal microscopy (IVCM) data of basal epithelial cell density, sub-basal dendritic cell density, nerve density, and nerve tortuosity were also measured (72). IVCM is used to identify structural features that are pathognomonic for DES, including decreased number of corneal epithelial cells, increased number of dendritic cells, reduced sub-basal nerve density, and increased nerve tortuosity (97).

There were no adverse events in either treatment group over the 3 months. The VB12 group showed statistically significant improvements in all clinical measurements and signs of DES, apart from nerve tortuosity, over the 3-month treatment. For the OXT group, all clinical and IVCM data showed significant improvement at 1 month, except for nerve tortuosity. Between the 1-month and 3-month timepoints, there were significant improvements in OSDI, TMH, BUT, and sub-basal nerve density; however, nerve tortuosity was increased. Like the VB12 group, the oxytocin group showed significant improvements at 3 months compared to baseline, except for nerve tortuosity and BUT. The OXT group IVCM data showed an increased basal epithelial cell density, decreased dendritic cell density, and increased sub-basal nerve density and tortuosity at 1 and 3 months, reflecting an overall improvement in DES conditions, apart from the increased nerve tortuosity (72). This study demonstrates a promising potential use of nebulized oxytocin as an effective treatment for DES.

## Conclusion

**Figure 1:**
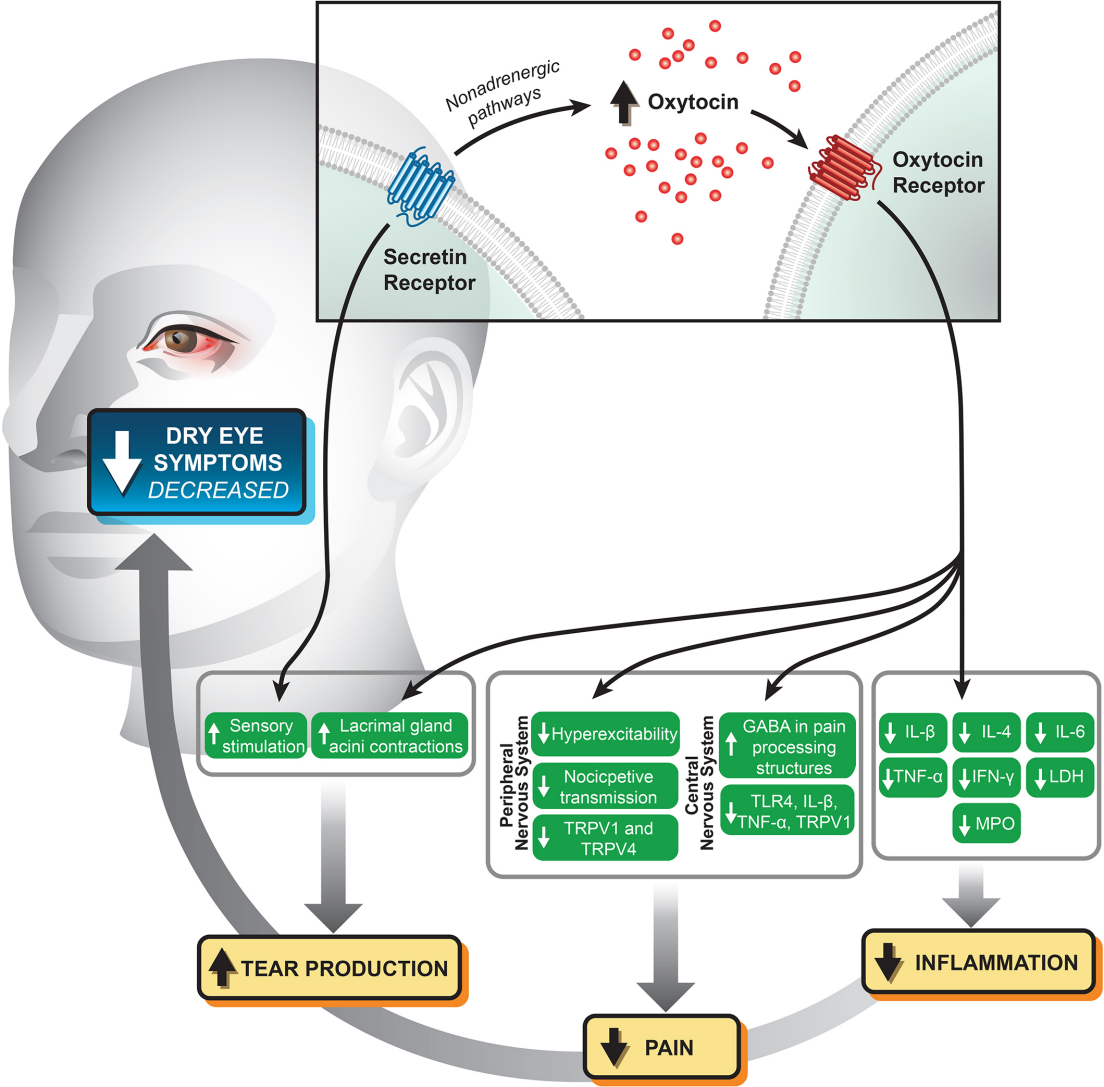
Potential mechanistic relationships and biomarkers relating stimulation of the oxytocin and secretin receptors at the ocular surface with modulation of pain and inflammation.

There is a growing body of evidence supporting the role of oxytocin and secretin in ocular surface homeostasis, outlined in **Figure 1**. Oxytocin promotes tear production by the lacrimal gland and appears to reduce pain *via* both neuronal and central mechanisms; its intranasal use has shown promise in the treatment of trigeminal pain. There is compelling evidence showing the anti-inflammatory and anti-nociceptive potential of oxytocin, especially in synergism with secretin, in intestinal mucosa and other tissue types. There may be a similar role for these peptide hormones in reducing ocular surface inflammatory and pain syndromes. Further studies are necessary to determine the physiologic functions of ocular surface oxytocin and secretin receptors and how stimulation of these receptors modulate inflammatory and pain pathways.

## Author contributions

All authors made substantial contributions to the conception of this mini-review and drafted or revised it critically for important intellectual content. They provide approval for publication of the content and agree to be accountable for all aspects of the work in ensuring that questions related to the accuracy or integrity of any part of the work are appropriately investigated and resolved.

## Funding

This review was supported by funds from the National Institutes of Health (NIH-NEI P30 EY002162 – Core Grant for Vision Research; and R01EY032161 awarded to M.F.C.), Research to Prevent Blindness (an unrestricted grant to the UCSF Department of Ophthalmology), and All May See Foundation.

## Acknowledgments

Thank you to Suling Wang and the UCSF Vision Core for assistance with creating the figure.

## Conflict of interest

BW along with Columbia University filed a patent application for the use of oxytocin and secretin for the treatment of ocular surface disease.

## Publisher’s note

All claims expressed in this article are solely those of the authors and do not necessarily represent those of their affiliated organizations, or those of the publisher, the editors and the reviewers. Any product that may be evaluated in this article, or claim that may be made by its manufacturer, is not guaranteed or endorsed by the publisher.
